# Unlocking a Ferrocenium Superoxidizer with the Perfluorinated Cp* Ligand

**DOI:** 10.1002/anie.202505783

**Published:** 2025-06-16

**Authors:** Robin Sievers, Nico G. Kub, Tim‐Niclas Streit, Marc Reimann, Günther Thiele, Martin Kaupp, Moritz Malischewski

**Affiliations:** ^1^ Institute of Chemistry and Biochemistry Freie Universität Berlin Fabeckstr. 34/36 14195 Berlin Germany; ^2^ Institute of Chemistry Technische Universität Berlin Str. des 17. Juni 115 10623 Berlin Germany

**Keywords:** Cyclopentadienyl ligands, Fluorinated ligands, Metallocenes, Organometallic compounds, Oxidations

## Abstract

The photolytically induced arene displacement of [Fe(C_5_H_5_)(*o*DCB)][PF_6_] (oDCB = *ortho*‐dichlorobenzene) in the presence of [NEt_4_][C_5_(CF_3_)_5_] afforded the highly fluorinated and benchstable ferrocene [Fe(C_5_H_5_)(C_5_(CF_3_)_5_)]. The perfluorinated Cp* ligand exerts an extreme electron withdrawing effect on the ferrocene with *E*_1/2_ = 1.35 V (versus Fc/Fc^+^). This proved to be the highest value obtained for any ferrocene reported so far. The corresponding stable and storable ferrocenium complex [Fe(C_5_H_5_)(C_5_(CF_3_)_5_)][AsF_6_] was generated in quantitative yield and represents not only the most oxidizing ferrocenium species, but also the strongest known isolable organometallic oxidizer. Its strength was demonstrated by the twofold oxidation of [Fe(C_5_(CH_3_)_5_)_2_] to its dication and an oxidative C‐H activation of *ortho*‐terphenyl. This unprecedented redox chemistry combined with perfluorocarbon solubility allows for selective and quantitative recycling of the highly fluorinated ferrocene. Together with the low basicity and inertness of [Fe(C_5_H_5_)(C_5_(CF_3_)_5_)], the chemistry of strong oxidizers is herein expanded into organometallics.

## Graphical abstract

The synthesis and full characterization of the electron deficient ferrocene [Fe(C_5_H_5_)(C_5_(CF_3_)_5_)] and its ferrocenium salt is reported. An extreme redox potential of *E*_1/2_ = 1.35 V (versus Fc/Fc^+^) enables its synthetic use as a powerful oxidizing agent.

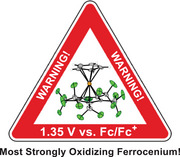

Once discovered by serendipity in 1951,^[^ ^1^, ^2^ ^]^ the iconic ferrocene (Fc) is nowadays described as a “major milestone in the development of chemistry” and inspires chemists from diverse fields such as (bio)organometallics, catalysis, polymers, and materials science by its unique properties.^[^ ^3^, ^4^, ^5^, ^6^ ^]^ This is not least due to its widely studied redox chemistry, involving the reversible one‐electron oxidation towards ferrocenium (Fc^+^).^[^ ^7^ ^]^ Therefore, the electronic structure and redox properties of ferrocene have been extensively tuned by different substitution patterns on the cyclopentadienyls (Cp) in various studies. Recent progress even allowed the unprecedented isolation of a ferrocene dication [Fe(C_5_(CH_3_)_5_)_2_]^2+^,^[^ ^8^, ^9^ ^]^ or ferrocene anion [Fe(C_5_ *^t^* Bu_3_H_2_)_2_]^−^.^[^ ^10^, ^11^, ^12^ ^]^

While electron‐rich ferrocene derivatives are now widely available, their electron‐deficient counterparts remain scarce. Nevertheless, electron deficient ferrocenes are in high demand by redox and material chemists alike,^[^ ^13^, ^14^, ^15^ ^]^ due to their pronounced oxidative stability. While the synthesis of such electron deficient ferrocenes has occasionally been achieved by the introduction of carboxyl and sulfonyl groups,^[^ ^16^, ^17^, ^18^ ^]^ halogenation proves superior, because of the associated chemical inertness. Although some halogenated ferrocenes,^[^ ^19^, ^20^, ^21^ ^]^ including the perhalogenated [Fe(C_5_Br_5_)_2_]^[^ ^22^ ^]^ and [Fe(C_5_Cl_5_)_2_], have been isolated,^[^ ^23^, ^24^ ^]^ fluorination appears most promising due to the resulting oxidative stability and applicability.^[^ ^25^ ^]^ However, the introduction of fluorine‐containing substituents proved to be synthetically challenging and it was not until 1992 that Hughes et al. reported in seminal work the first transition metal complex [Ru(C_5_(CH_3_)_5_)(C_5_F_5_)] containing a perfluorocyclopentadienyl ligand.^[^ ^26^, ^27^ ^]^ In 2015, Sünkel's group continued with an impressive synthesis of [Fe(C_5_H_5_)(C_5_F_5_)] by stepwise electrophilic fluorination.^[^ ^28^ ^]^ Surprisingly, the effect of direct fluorination on the oxidative stability is only moderate with a redox potential of *E*_1/2_ = 0.82 V (versus Fc/Fc^+^) for [Fe(C_5_H_5_)(C_5_F_5_)],^[^ ^29^ ^]^ which is mainly explained by the pronounced + M‐effect of fluorine. Notably, no stable corresponding ferrocenium [Fe(C_5_H_5_)(C_5_F_5_)]^+^ was reported. In contrast, the introduction of trifluoromethyl groups is expected to result in significant electron withdrawal, due to the absence of most conjugative donor effects. This trend can be clearly seen in the oxidation potentials of the known 1,1´‐disubstituted ferrocenes [Fe(C_5_H_4_R)_2_] (R = F, CF_3_) with an *E*_1/2_ = 0.22 V and 0.64 V (versus Fc/Fc^+^) respectively^[^ ^30^, ^31^, ^32^ ^]^ (Figure 1). Despite remarkable progress in the field of electrophilic trifluoromethylation by groups such as Togni et al.,^[^ ^33^, ^34^, ^35^, ^36^, ^37^ ^]^ ferrocene remains a challenging substrate.^[^ ^38^, ^39^, ^40^ ^]^

**Figure 1:**
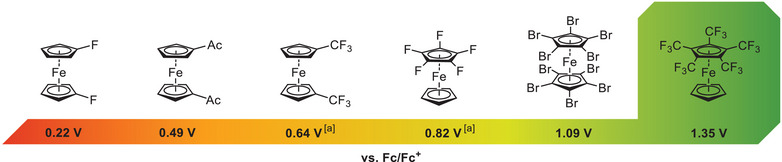
Selected electron deficient ferrocenes with their respective *E_1/2_* redox potentials (versus Fc/Fc^+^).^[^ ^18^, ^22^, ^29^, ^32^ ^]^ a) Ferrocenium salt not reported.

However, the perfluorinated Cp* ([C_5_(CF_3_)_5_]^−^) has been known since 1980,^[^ ^41^, ^42^, ^43^ ^]^ and more recently we were able to introduce it as a ligand in coordination chemistry,^[^ ^44^, ^45^, ^46^, ^47^, ^48^ ^]^ thus making it a suitable candidate for the synthesis of an exceptionally electron‐poor and oxidatively stable metallocene. Assuming a linear increase of *E*_1/2_ (as observed for F) for each CF_3_ group,^[^ ^28^ ^]^ the by far highest oxidation potential for a ferrocene would result. Consequently, the formation of [Fe(C_5_H_5_)(C_5_(CF_3_)_5_)]^+^ could yield the strongest oxidizing ferrocenium known to date (even surpassing former strongly oxidizing organometallics like the dicationic [Ni(C_5_H_5_)_2_]^2+^ with *E*_1/2_ = 1.17 V (versus Fc/Fc^+^),^[^ ^49^ ^]^ making it a potential superoxidizer. Such strong oxidizers are in demand, but currently limited to recent advances by Krossing et al. with their fluorinated organic radical cations.^[^ ^50^, ^51^, ^52^, ^53^ ^]^ Here, [Fe(C_5_H_5_)(C_5_(CF_3_)_5_)]^+^ could be an unprecedented extension into organometallics, allowing for example the selective generation and isolation of highly reactive cations.

For this purpose, the synthesis of [Fe(C_5_H_5_)(C_5_(CF_3_)_5_)] was performed by a photolytic (470 nm) arene displacement of the literature‐known [Fe(C_5_H_5_)(*o*DCB)][PF_6_] (*o*DCB = *ortho*‐dichlorobenzene) complex in the presence of [NEt_4_][C_5_(CF_3_)_5_] (Scheme 1).^[^ ^54^ ^]^ Due to the weak binding character of [C_5_(CF_3_)_5_]^−^, this conversion is only feasible with significantly electron‐poor arenes, such as *o*DCB, while electron‐rich derivatives (e.g., [Fe(C_5_H_5_)(C_6_H_6_)][PF_6_]) do not show the analogous reactivity. Similarly, the choice of the solvent proved crucial, as coordinating solvents (e.g., THF, MeCN, PhMe) hindered any coordination of the perfluorinated Cp*, and weakly‐basic solvents such as CH_2_Cl_2_ led to significant decomposition. Only the reaction in *o*DFB (*o*DFB = *ortho*‐difluorobenzene) gave the desired ferrocene [Fe(C_5_H_5_)(C_5_(CF_3_)_5_)] in 60% yield. This is probably explained by an in situ formation of the highly reactive intermediate [Fe(C_5_H_5_)(*o*DFB)]^+^, which subsequently undergoes the coordination of the perfluorinated Cp*. Quantum chemical calculations (ωB97X‐D4/def2‐QZVPPD//r^2^SCAN‐3c level) suggest that binding of *o*DCB is preferred over binding of *o*DFB by about 3 kJ mol^−1^, which would indeed make the proposed intermediate even more reactive.

**Scheme 1:**
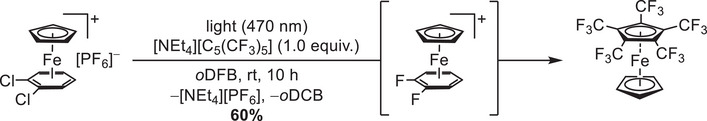
Synthesis of [Fe(C_5_H_5_)(C_5_(CF_3_)_5_)] by photolytic arene displacement of [Fe(C_5_H_5_)(*o*DCB)][PF_6_] in *o*DFB.

The highly fluorinated ferrocene [Fe(C_5_H_5_)(C_5_(CF_3_)_5_)] appears as a volatile greenish solid, that is completely stable at ambient conditions. While other highly halogenated ferrocenes (e.g., [Fe(C_5_Br_5_)_2_] and [Fe(C_5_Cl_5_)_2_]) suffer from low solubility, [Fe(C_5_H_5_)(C_5_(CF_3_)_5_)] exhibits a general high solubility in organic solvents. In addition, solubility in the fluorous‐phase was observed which is highly unusual for metallocenes, allowing potential applications in perfluorocarbons and selective recovery from reaction mixtures.^[^ ^45^, ^55^, ^56^, ^57^, ^58^, ^59^ ^]^ To explain the significant color shift compared to the usual orange of ferrocenes, a UV–vis spectrum was measured in CH_2_Cl_2_. For [Fe(C_5_H_5_)_2_] the absorption maximum is reported at 441 nm, resulting from the spin‐allowed ^1^A_1g_ → ^1^E_1g_ and ^1^A_1g_ → ^1^E_2g_ d‐d transitions.^[^ ^60^, ^61^ ^]^ While most substitution patterns lead to a distinct red shift, a hypsochromic shift is observed for [Fe(C_5_H_5_)(C_5_(CF_3_)_5_)], giving a value of 407 nm (see Figure S17 for the UV–vis spectrum). Similar effects have already been observed for fluoroferrocenes [Fe(C_5_H_5_)(C_5_H_5‐n_F_n_)] (*n* = 1–5) with shifts down to 395 nm.^[^ ^29^ ^]^ A matching trend can be obtained from calculated orbital energy differences between the corresponding d‐orbitals (ωB97X‐D4/def2‐QZVPPD//r^2^SCAN‐3c level) which are increased from 10.46 eV ([Fe(C_5_H_5_)_2_]) to 10.93 eV ([Fe(C_5_H_5_)(C_5_F_5_)]) and 10.72 eV ([Fe(C_5_H_5_)(C_5_(CF_3_)_5_)]), respectively. The smaller gap in the [C_5_(CF_3_)_5_]^−^ system compared to [Fe(C_5_H_5_)(C_5_F_5_)] is due to some compensatory effects, as occupied and virtual d‐orbitals are stabilized by about 0.7 and 0.9 eV, respectively, by the [C_5_(CF_3_)_5_]^−^ ligand compared to pentafluoroferrocene. Compared to [Fe(C_5_H_5_)_2_], the [C_5_F_5_]^−^ ligand stabilizes the occupied and unoccupied d‐orbitals by 0.9 and 0.4 eV, respectively, while [Fe(C_5_H_5_)(C_5_(CF_3_)_5_)] shows much more similar stabilization for both sets of orbitals relative to [Fe(C_5_H_5_)_2_] (1.5 and 1.3 eV, respectively). The shape of the frontier orbitals is almost identical for all three ferrocenes, being dominated by Fe d‐orbitals (the frontier orbitals of [Fe(C_5_H_5_)(C_5_(CF_3_)_5_)] are explicitly shown in Figure S22). We note in passing that for [Fe(C_5_H_5_)_2_] and [Fe(C_5_H_5_)(C_5_F_5_)], the lowest‐lying unoccupied MOs do not correspond to the metal 3d‐orbitals at the chosen level of theory. The d‐orbitals are very slightly higher in these cases. [Fe(C_5_H_5_)(C_5_(CF_3_)_5_)] was characterized by NMR spectroscopy in CD_2_Cl_2_. The ^1^H NMR spectrum shows a singlet at 4.94 ppm, which constitutes a significant low‐field shift relative to the parent unsubstituted ferrocene (δ = 4.16 ppm). The same trend is seen in the ^13^C{^1^H} NMR spectrum with a singlet at 78.1 ppm ([Fe(C_5_H_5_)_2_]: *δ* = 68.3 ppm). Both values emphasize the extreme electron withdrawal from the ferrocene exerted by the perfluorinated Cp* ligand. Corresponding carbon resonances of the [C_5_(CF_3_)_5_]^−^ ligand are only observed in the ^13^C{^19^F} NMR spectrum with a multiplet at 123.3 and a singlet at 112.5 ppm, for the trifluoromethyl groups and the C_5_‐moiety, respectively. The ^19^F NMR spectrum shows a very small low field shift compared to ionic [C_5_(CF_3_)_5_]^−^ (*δ* = −50.6 ppm), giving a singlet at −50.3 ppm.

Single crystals were obtained from solution in perfluorohexanes, by slow cooling to −70 °C. [Fe(C_5_H_5_)(C_5_(CF_3_)_5_)] crystallizes in the monoclinic *P*2_1_ space group and shows a coplanar *η* ^5^‐coordination towards both Cp ligands (Figure 2). While regular ferrocene has a Fe‐Cp_centroid_ distance of 1.65 Å,^[^ ^62^ ^]^ values of 1.678(1) and 1.623(1) Å are observed for [C_5_H_5_]^−^ and [C_5_(CF_3_)_5_]^−,^ respectively. At first sight this is somewhat surprising, as the perfluorinated Cp* ligand is known to coordinate weakly towards metal centers, which was demonstrated in the substitution lability of [Rh(COD)(C_5_(CF_3_)_5_)].^[^ ^44^, ^45^, ^46^, ^47^ ^]^ Quantum chemical calculations at the r^2^SCAN‐3c level confirm this trend, giving 1.665 and 1.613 Å, respectively, compared to 1.646 Å in regular ferrocene. Energy decomposition analysis results (see Table S3), on the other hand, also confirm the significantly smaller interaction between the metal center and the [C_5_(CF_3_)_5_]^−^ ligand (433 kJ mol^−1^) compared to the [C_5_H_5_]^−^ ligand (698 kJ mol^−1^). Interestingly, this last interaction energy is significantly larger than in native ferrocene (555 kJ mol^−1^) and in pentafluoroferrocene (572 kJ mol^−1^). This is a result of the strong push‐pull nature of [Fe(C_5_H_5_)(C_5_(CF_3_)_5_)], which can also be inferred from its sizable dipole moment (5.0 D at r^2^SCAN‐3c level). The increase in interaction energy can be traced back to the increase in π‐bonding between Fe and [C_5_H_5_]^−^ (see Tables S3, S4), most likely due to the stabilization of the unoccupied Fe d‐orbitals by the [C_5_(CF_3_)_5_]^−^ ligand. The push‐pull character seems to also be responsible for the increased Fe‐Cp distance in [Fe(C_5_H_5_)(C_5_(CF_3_)_5_)]: A distance similar to that in [Fe(C_5_H_5_)_2_)] decreases the dipole moment of the compound. In our model, this leads to a destabilization by the dielectric environment (corresponding to either the crystal or the solvent), which cannot be compensated by a larger local stabilization (see Table S3).

**Figure 2:**
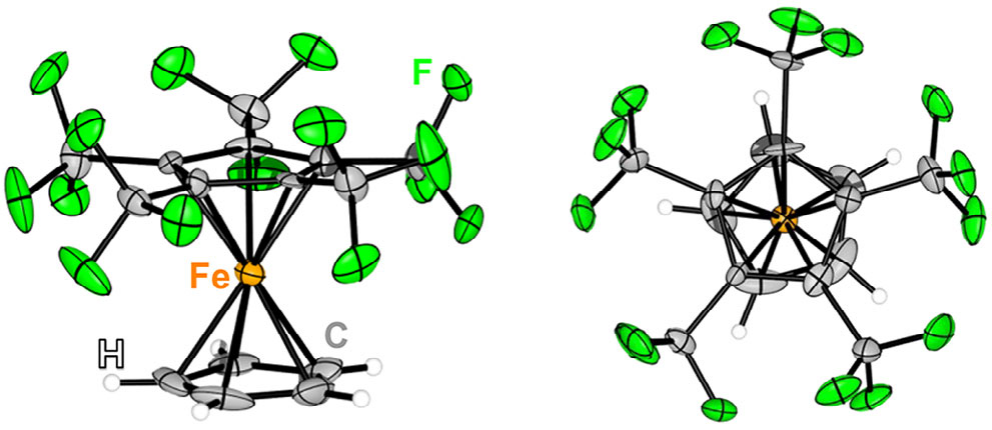
Molecular structure in the solid state of [Fe(C_5_H_5_)(C_5_(CF_3_)_5_)] with side view (left) and top view (right). Ellipsoids are depicted with 50% probability level. Color code: white—hydrogen, grey—carbon, green—fluorine, orange—iron.

The redox chemistry of the extremely electron deficient ferrocene [Fe(C_5_H_5_)(C_5_(CF_3_)_5_)] has been investigated by cyclic voltammetry. A quasi‐reversible one‐electron oxidation process was observed in hexafluoroisopropanol (HFIP) with [*n*Bu_4_N][PF_6_] as a supporting electrolyte (Figure 3). The half‐wave potential was determined to be *E*_1/2_ = 1.35 V with a peak oxidation potential of *E*_pa_ = 1.50 V (versus Fc/Fc^+^), which is the highest value obtained so far for any ferrocene. These values are in excellent agreement with our quantum‐chemically calculated value of *E*_1/2_ = 1.45 V (versus Fc/Fc^+^, see Table S5). Inspection of the calculated spin‐density shows a purely iron based oxidation, virtually identical to other ferrocene derivatives. In other organic solvents besides HFIP (e.g., CH_2_Cl_2_ or *o*DFB) the oxidation process appeared to be irreversible, but showed even higher peak potentials up to *E*_pa_ = 1.70 V (see Figure S19). This shows a solvent dependence for [Fe(C_5_H_5_)(C_5_(CF_3_)_5_)] as the oxidation potential increases in less polar solvents (compared to HFIP). In general, these potentials are almost twice the value of the pentafluoroferrocene with *E*_1/2_ = 0.82 V (versus Fc/Fc^+^).^[^ ^29^ ^]^ The more pronounced electron withdrawal by trifluoromethylation compared to direct fluorination on a Cp ligand is clearly apparent, due to a lack of resonance effects with the π‐system. The strongly electron‐withdrawing effect of the CF_3_ groups is also reflected by a quasi‐reversible reduction of [Fe(C_5_H_5_)(C_5_(CF_3_)_5_)] in THF at *E*_1/2_ = −2.2 V (see Figure S18).

**Figure 3:**
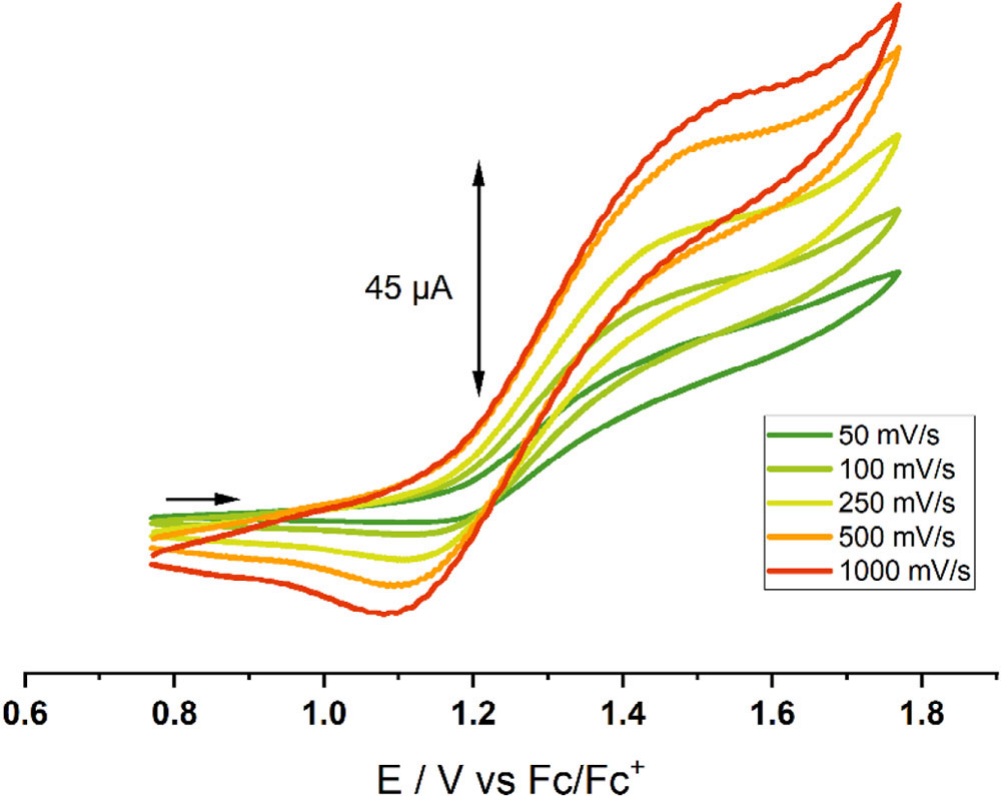
Cyclic voltammogram of [Fe(C_5_H_5_)(C_5_(CF_3_)_5_)] showing a quasi‐reversible oxidation in HFIP with [*n*Bu_4_N][PF_6_] as a supporting electrolyte.

For previous halogenated ferrocenes, such as [Fe(C_5_H_4_(CF_3_))_2_],^[^ ^32^ ^]^ [Fe(C_5_H_5_)(C_5_F_5_)]^[^ ^28^ ^]^ and [Fe(C_5_Cl_5_)_2_]^[^ ^23^, ^24^ ^]^ oxidation and isolation of the corresponding ferrocenium salts was either hindered by decomposition or seemingly not investigated. In contrast, the quantitative oxidation of [Fe(C_5_H_5_)(C_5_(CF_3_)_5_)] was possible by reaction with AsF_5_ in liquid SO_2_ (Scheme 2), yielding the corresponding ferrocenium [Fe(C_5_H_5_)(C_5_(CF_3_)_5_)][AsF_6_] (and AsF_3_ as a volatile side product) within 5 min. Single crystals suitable for XRD were obtained from solution in *a*HF by slow cooling from −20 to −70 °C, confirming the identity of the ferrocenium salt as [Fe(C_5_H_5_)(C_5_(CF_3_)_5_)][AsF_6_] · 0.33 AsF_3_ (see Table S2 and Figure S21 for molecular structure in solid state). Surprisingly, the deep green [Fe(C_5_H_5_)(C_5_(CF_3_)_5_)][AsF_6_] is stable and storable at room temperature and can also be handled in common aprotic organic solvents. With the mentioned anodic peak potentials up to 1.70 V (versus Fc/Fc^+^) it is the most oxidizing ferrocenium species known. Since Connelly and Geiger categorized oxidants with redox potentials > 0.8 V as “very strong” ^[^ ^18^ ^]^ we suggest the term “superoxidizer” for reagents with half‐wave potentials of *E*_1/2_ ≥ 1.0 V. Furthermore, to the best of our knowledge, [Fe(C_5_H_5_)(C_5_(CF_3_)_5_)][AsF_6_] is the strongest isolable organometallic oxidizer to date.

**Scheme 2:**
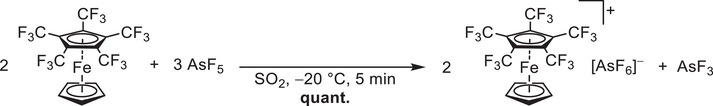
Synthesis of [Fe(C_5_H_5_)(C_5_(CF_3_)_5_)][AsF_6_] by oxidation with AsF_5_ in liquid SO_2_.

To demonstrate the oxidative power of [Fe(C_5_H_5_)(C_5_(CF_3_)_5_)][AsF_6_], the reaction with permethylated [Fe(C_5_(CH_3_)_5_)_2_] was carried out (Scheme 3). Here, even a twofold oxidation to the literature‐known orange‐brown dication [Fe(C_5_(CH_3_)_5_)_2_][AsF_6_]_2_ (*E*_pa_ = 1.23 V in SO_2_)^[^ ^63^ ^]^ was observed by single crystal X‐ray diffraction. Interestingly, the Fe^+II^ center of [Fe(C_5_H_5_)(C_5_(CF_3_)_5_)] can withstand the presence of Fe^+IV^ without any comproportionation towards Fe^+III^, due to its oxidative resistance. Notably, [Fe(C_5_(CH_3_)_5_)_2_]^2+^ was so far only accessible by using very strong inorganic oxidizers since even reactions with potent organic radical cations failed.^[^ ^8^, ^50^ ^]^

**Scheme 3:**
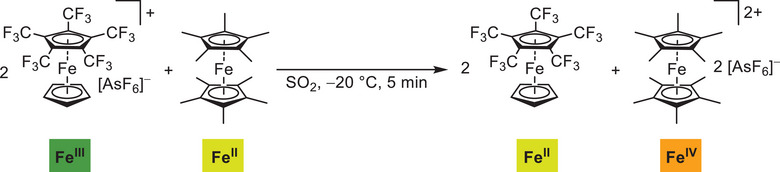
Twofold oxidation of [Fe(C_5_(CH_3_)_5_)_2_] to its dication by [Fe(C_5_H_5_)(C_5_(CF_3_)_5_)][AsF_6_].

Another example is the reaction of [Fe(C_5_H_5_)(C_5_(CF_3_)_5_)][AsF_6_] with a two‐fold methoxy‐substituted *ortho*‐terphenyl in *o*DFB (Scheme 4). This gives the corresponding triphenylene in almost quantitative yield by oxidative C–C coupling analogous to the Scholl reaction (presumably accompanied by HF/AsF_5_ formation). Comparable oxidative arene couplings usually require electron rich or activated substrates and strong inorganic oxidizers as for example MoCl_5_.^[^ ^64^ ^]^ In both oxidation examples the reduced [Fe(C_5_H_5_)(C_5_(CF_3_)_5_)] could be recovered from the reaction mixture by selective extraction with perfluorohexanes. This enables simple purification procedures, allowing for subsequent regeneration of the oxidizer and inspiring unique biphasic applications.

**Scheme 4:**
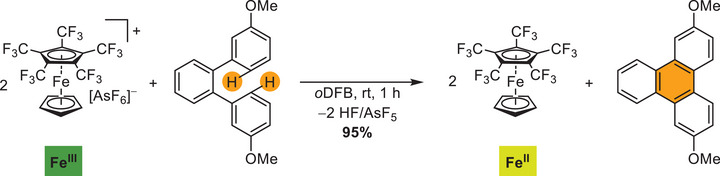
Oxidative C–C coupling of *ortho*‐terphenyl to triphenylene by [Fe(C_5_H_5_)(C_5_(CF_3_)_5_)][AsF_6_].

In summary the synthesis and characterization of the extremely electron‐deficient and oxidatively stable ferrocene [Fe(C_5_H_5_)(C_5_(CF_3_)_5_)] is reported. Cyclic voltammetry revealed a quasi‐reversible oxidation process with a half‐wave potential of *E*_1/2_ = 1.35 V and peak potentials up to 1.70 V (versus Fc/Fc^+^), the highest values obtained so far for any ferrocene. Quantitative chemical oxidation to the stable and storable superoxidizer [Fe(C_5_H_5_)(C_5_(CF_3_)_5_)][AsF_6_] was possible, yielding the strongest isolable organometallic oxidizer known to date. Its oxidative power was demonstrated by the twofold oxidation of [Fe(C_5_(CH_3_)_5_)_2_] to its dication and an unprecedented oxidative C–H activation of *ortho*‐terphenyl. The perfluorocarbon solubility of [Fe(C_5_H_5_)(C_5_(CF_3_)_5_)] allows for selective purification and recycling. This extends the field of very strong oxidizers to organometallics and may offer interesting applications for both inorganic and organic chemists.

## Supporting Information

The authors have cited additional references within the Supporting Information.^[^ ^65^, ^66^, ^67^, ^68^, ^69^, ^70^, ^71^, ^72^, ^73^, ^74^, ^75^, ^76^, ^77^, ^78^, ^79^, ^80^, ^81^, ^82^, ^83^, ^84^, ^85^, ^86^, ^87^, ^88^, ^89^, ^90^ ^]^ Deposition numbers 2429414 (for fluorinated ferrocene) and 2429415 (for fluorinated ferrocenium) contain the supplementary crystallographic data for this paper. This data is provided free of charge by the joint Cambridge Crystallographic Data Centre and Fachinformationszentrum Karlsruhe Access Structures service www.ccdc.cam.ac.uk/structures. A preliminary version of this manuscript has been deposited on the preprint server *ChemRxiv*: https://doi.org/10.26434/chemrxiv‐2025‐lqllp.

## Conflict of Interests

The authors declare no conflict of interest.

## Supporting information



Supporting Information

## Data Availability

The data that support the findings of this study are available in the supplementary material of this article.
