# Supplementary material for: Unlocking a Ferrocenium Superoxidizer with the Perfluorinated Cp* Ligand
Source: Angew Chem Int Ed Engl. 2025 Jun 16;64(31):e202505783. doi: 10.1002/anie.202505783 (PMC12304867; doi:10.1002/anie.202505783)
Supplement: Supplementary file 1 — Supporting Information [file ANIE-64-e202505783-s001.docx]

**Unlocking a Ferrocenium Superoxidizer with the Perfluorinated Cp* Ligand**

Robin Sievers, Nico G. Kub, Tim-Niclas Streit, Marc Reimann, Günther Thiele, Martin Kaupp, Moritz Malischewski*

[a] R. Sievers, N. G. Kub, T.-N. Streit, Dr. G. Thiele, Dr. M. Malischewski
Institute of Chemistry and Biochemistry
Freie Universität Berlin
Fabeckstr. 34/36, 14195 Berlin, Germany
E-mail: moritz.malischewski@fu-berlin.de

[b] Dr. M. Reimann, Prof. Dr. M. Kaupp
Institute of Chemistry
Technische Universität Berlin
Str. des 17. Juni 115, 10623 Berlin

**Contents**

[General Information 2](#_Toc192416453)

[Synthetic Procedures 5](#_Toc192416454)

[NMR Spectra 9](#_Toc192416455)

[IR Spectra 16](#_Toc192416456)

[UV/VIS Spectra 17](#_Toc192416457)

[Cyclic Voltammograms 17](#_Toc192416458)

[Crystallographic Data 17](#_Toc192416459)

[Quantum Chemical Calculations 23](#_Toc192416460)

[References 25](#_Toc192416461)

# General Information

Reactions and workups sensitive to air were performed in previously heated glassware under an atmosphere of argon using standard Schlenk techniques and an oil pump vacuum of 10^−3^ mbar. Room temperature (rt) refers to 25 °C. The addition of liquid reagents and solvents was done by using threefold argon-flushed disposable syringes and septa, while solids were added in argon stream. Low temperature reactions were performed in a cooled ethanol-bath. Glassware was cleaned by storing in a potassium hydroxide bath for several days, rinsed with diluted hydrochloric acid and doubly deionized water and dried at 150 °C.

**Pressure reactions**

The synthesis of [NEt_4_][C_5_(CF_3_)_5_] involves high temperatures and highly volatile substances in a closed system. Hence, it must be assumed, that high pressures arise upon heating and advanced caution is required. Therefore, it is advisable to perform the reaction in a separate and properly closed fumehood. The thick-walled glass reaction vessel should not be opened and if possible, not even touched until the reaction has finished and reached rt.

**Solvents, reagents and materials**

Anhydrous MeCN and CH_2_Cl_2_ were obtained from the solvent system FMBRAUN MB SPS-800 and stored over activated 3 Å mol sieves. Anhydrous *o*DCB and *o*DFB were used as purchased and stored over activated 3 Å mol sieves. SO_2_ was used as purchased and stored over CaH_2_. Anhydrous *a*HF was dried with elemental F_2_. Gaseous arsenic pentafluoride was prepared by reaction of elemental arsenium with elemental fluorine. SO_2_, *a*HF and AsF_5_ are toxic gases at room temperatures and were stored in stainless steel cylinders. Deuterated solvents CD_2_Cl_2_, CDCl_3_ and (CD_3_)_2_CO were used as purchased and stored over activated 3 Å mol sieves. Sulfolane was heated at 60 °C for at least 24 h over activated 3 Å mol sieves and additionally 1 h in high vacuum prior to use. 18-crown-6 was heated at 80 °C for 2 h in high vacuum prior to use. All other solvents and commercially available reagents were used without further purification. Photochemistry was performed with a blue LED (470 nm) from OSRAM mounted on a 10 cm^3^ aluminum heat sink. PFA tube (8 mm) chemistry was performed at a steel line from SWADGELOK.

**Nuclear magnetic resonance (NMR) spectroscopy**

NMR spectroscopy was measured on a JEOL ECX 400 (400 MHz) or a Varian INOVA 600 (600 MHz) in the reported deuterated solvents CD_2_Cl_2_, CDCl_3_ and (CD_3_)_2_CO. All given chemical shifts in ^1^H NMR spectra are calibrated on the resonance signals of CHCl_3_ contained in CDCl_3_ (*δ* = 7.26 ppm), CDHCl_2_ contained in CD_2_Cl_2_ (*δ* = 5.32 ppm) and (CHD_2_)(CD_3_)CO (*δ* = 2.05 ppm) contained in (CD_3_)_2_CO. The ^13^C NMR spectra are calibrated on the respective resonance signals of CDCl_3_ (*δ* = 77.16 ppm), CD_2_Cl_2_ (*δ* = 53.84 ppm) and (CD_3_)_2_CO (*δ* = 29.84 and 206.26 ppm).^[65,66]^ The ^19^F and ^31^P NMR spectra are device-internally calibrated relative to the resonance signal of CFCl_3_ and H_3_PO_4_ according to the unified chemical shift scale.^[67]^ The given multiplicities are phenomenological, thus the actual appearance of the signals is stated and not the theoretically expected one. The following abbreviations were used and analogously combined to designate multiplicities: s (singlet), d (doublet), t (triplet), q (quartet), m (multiplet), m_c_ (centrosymmetric multiplet). For centrosymmetric multiplets the center and for non-symmetric multiplets the interval is stated. Evaluation of spectra was performed with Mestrelab Research MNova 7.^[68]^

**Infrared (IR) spectroscopy**

IR spectroscopy was measured on a FT (Fourier transformation) Nicolet iS10 IR-spectrometer. The sample was directly measured by ATR (attenuated total reflection) technique. Characteristic absorptions are given in wavenumbers *ṽ* [cm^‑1^] and intensities are stated as vs (very strong), s (strong), m (medium) and w (weak).

**High resolution mass spectrometry (HRMS) and elemental analysis (EA)**

HRMS was recorded using a VARIAN MAT 711 by electron impact ionization (EI) at the department of mass spectrometry at the Freie Universität Berlin. A detailed listing of fragmentation is dispensed, instead the molecular ion peak or a characteristic fragment peak is stated. EA was measured on a VARIO EL. Relative proportion of C and H are given in percent.

**Ultraviolet and visible light (UV/VIS) spectroscopy**

UV/VIS spectra were recorded on a PerkinElmer Lambda 465 photometer with deuterium and tungsten lamps. A fiber optic probe was used to record the spectra.

**X-ray diffraction (XRD)**

X-Ray data were collected on a BRUKER D8 Venture system. Data were collected at 100(2) K using graphite monochromated Mo K_α_ radiation (*λ*_α_ = 0.71073 Å). The strategy for the data collection was evaluated by using the Smart software. The data were collected by the standard “*ψ*-*ω* scan techniques” and were scaled and reduced using Saint+software. The structures were solved by using Olex2,^[69]^ the structure was solved with the XT^[70]^ structure solution program using Intrinsic Phasing and refined with the XL refinement package^[71,72]^ using Least Squares minimization. Bond length and angles were measured with Diamond Crystal and Molecular Structure Visualization Version 4.6.2.^[73]^ Drawings were generated with POV-Ray.^[74]^

**Quantum chemical calculations**

Molecular structures have been optimized at the r^2^SCAN-3c level^[75]^ using the ORCA program, version 6.0.0.^[76]^ All calculations included a continuum solvent model (CPCM with parametrization for CH_2_Cl_2_ as a solvent with intermediate polarity) to approximately account for the dielectric effects of different solvents or the crystal environment. Computations used a tight DFT grid (DefGrid3), and tight optimization criteria (VeryTightSCF and VeryTightOpt). Harmonic vibrational frequency calculations confirmed that all structures are minima on the potential energy surface. Electrochemical potentials against the ferrocene/ferrocenium electrode were performed using additional gas phase single points at the ωB97X-D4^[77]^ level to extract electronic energy contributions, using def2-QZVPPD^[78]^ basis sets and the RIJCOSX approximation.^[79]^ Solvation effects were estimated by ORCA’s implementation of the COSMO-RS approach,^[80]^ also using CH_2_Cl_2_ as a solvent. Intramolecular free energy contributions were included using Grimme’s modified rigid-rotor, harmonic-oscillator approach.^[81]^ Energy decomposition analyses and extended transition-state analyses with natural orbitals for chemical valence (ETS-NOCV) were performed at the BP86+D4/TZP^[82-85]^ level using the ADF engine of the AMS program package, release 2023.1.^[86,87]^

# Synthetic Procedures

**Reaction S1: [NEt_4_][C_5_(CF_3_)_5_]**

In a dried 1000 mL pressure flask anhydrous KF (60 g, 1.0 mol, 11 equiv.) was placed in anhydrous and degassed sulfolane (190 mL) under an atmosphere of argon. Anhydrous and degassed 18-crown-6 (8.7 g, 33 mmol, 0.33 equiv.) and hexachlorobuta-1,3-diene (15 mL, 96 mmol, 1.0 equiv.) were added at rt. The resulting reaction mixture was carefully shaken and cooled to −196 °C in high vacuum. The properly closed pressure flask was slowly warmed to 190 °C and stirred at this temperature for 3 d. Then the resulting black suspension was cooled to rt and the volatiles were removed in high vacuum. The remaining mixture was filtrated under an atmosphere of argon and the residue was extracted with anhydrous MeCN (3 × 40 mL). The filtrate was warmed to 40 °C and all MeCN was removed in high vacuum, while stirring. The resulting solution was put under high vacuum and H_2_SO_4_ (conc., 200 mL, 3.6 mol, 38 equiv.) was added dropwise at rt over a period of 3 h, while stirring and continuously collecting the volatiles in a cold trap of −196 °C. After complete addition, the mixture remained for additional 2 h in high vacuum. The cold trap was put under argon and slowly warmed to 0 °C, giving a pale yellow liquid. Then CH_2_Cl_2_ (20 mL) and a solution of [NEt_4_][OH] (35% in water, 10 mL, 24 mmol, 0.25 equiv.) were added and the reaction mixture was stirred for 15 min at rt, giving a deep red solution. The aqueous layer was separated and extracted with CH_2_Cl_2_ (4 × 20 mL). The combined organic layers were dried over MgSO_4_, filtrated and the solvent was removed under reduced pressure. The remaining solid was suspended in Et_2_O (~5 mL) and recrystallized twice from CH_2_Cl_2_ (~10 mL) by slowly cooling to −20 °C. The crystalline residue was decanted and washed with Et_2_O (2 × 5 mL). The solvents were removed under reduced pressure to give a product mixture of 85 mol% [NEt_4_][C_5_(CF_3_)_5_] (1.7 g, 3.2 mmol) and 15 mol% [NEt_4_][C_5_(CF_3_)_4_H] (0.30 g, 0.50 mmol) that was placed in a dried 50 mL Schlenk flask in anhydrous MeCN (20 mL). [NO][BF_4_] (0.26 g, 2.2 mmol, 0.60 equiv.) was added and the reaction mixture was stirred at room temperature for 4 h. Dest. H_2_O (20 mL) was slowly added and stirred for 15 min before separating aqueous and organic phase. The aqueous phase was extracted with CH_2_Cl_2_ (3 × 20 mL). The combined organic phases were dried over MgSO_4_, filtrated and the solvents removed under reduced pressure. The residue was dissolved in CH_2_Cl_2_ (1 mL) and slowly added to stirred *n*Bu_2_O (200 mL). The colorless suspension was filtrated and the residue washed with *n*Bu_2_O (2 × 10 mL) and n-pentane (2 × 10 mL). The solvent was removed under reduced pressure to give [NEt_4_][C_5_(CF_3_)_5_] (1.7 g, 3.2 mmol) as a colorless amorphous solid with a yield of 9%.

**^1^H NMR** (400 MHz, CD_2_Cl_2_, rt) *δ* [ppm] = 2.98 (q, ^3^*J*_H,H_ = 7.3 Hz, 8H), 1.21 (q, ^3^*J*_H,H_ = 7.1 Hz, 12H). **^19^F NMR** (377 MHz, CD_2_Cl_2_, rt) *δ* [ppm] = −50.6 (s, 15F). **^13^C{^1^H} NMR** (151 MHz, CD_2_Cl_2_, rt) *δ* [ppm] = 52.7 (m_c_, 4C), 7.3 (s, 4C). **^13^C{^19^F} NMR** (151 MHz, CD_2_Cl_2_, rt) *δ* [ppm] = 125.4 (s, 5C), 110.2 (s, 5C). The analytical data are consistent with those reported in literature.^[44,88]^

**Reaction S2: [Fe(C_5_H_5_)(*o*DCB)][PF_6_]**

In a dried 500 mL Schlenk flask [Fe(C_5_H_5_)_2_] (6.0 g, 32 mmol, 1.0 equiv.), powdered aluminium (0.72 g, 27 mmol, 0.80 equiv.) and AlCl_3_ (8.0 g, 60 mmol, 1.9 equiv.) were suspended in anhydrous *o*DCB (100 mL) under an atmosphere in argon. The reaction mixture was stirred under reflux for 12 h. The brownish suspension was cooled to 0 °C and dest. H_2_O (300 mL) was slowly added while stirring. The aqueous phase was isolated and washed with *n*-pentane (3 × 100 mL). A solution of LiPF_6_ (5.2 g, 36 mmol, 1.1 equiv.) in dest. H_2_O (40 mL) was added and the reaction mixture was stirred for 15 min. The resulting suspension was filtrated and the residue was washed with dest. H_2_O (3 × 40 mL). The crude solid was dissolved in acetone (5 mL) and slowly added to stirred Et_2_O (100 mL). The suspension was filtrated and the residue was washed with Et_2_O (3 × 40 mL) and dried under reduced pressure to give [Fe(C_5_H_5_)(*o*DCB)][PF_6_] (6.2 g, 15 mmol) as a greenish amorphous solid with a yield of 47%.

**^1^H NMR** (400 MHz, (CD_3_)_2_CO, rt) *δ* [ppm] = 7.07 (s, 2H), 6.61 (s, 2H), 5.39 (s, 5H).^[14]^ **^19^F NMR** (377 MHz, (CD_3_)_2_CO, rt) *δ* [ppm] = −72.2 (d, ^1^*J*_F,P_ = 708.1 Hz, 6F). **^31^P{^19^F} NMR** (162 MHz, (CD_3_)_2_CO, rt) *δ* [ppm] = −144.2 (s, 1P). **^13^C{^1^H} NMR** (100 MHz, (CD_3_)_2_CO, rt) *δ* [ppm] = 107.5 (s, 2C), 89.4 (s, 2C), 88.3 (s, 2C), 81.7 (s, 5C). The analytical data are consistent with those reported in literature.^[89]^

**Reaction S3: [Fe(C_5_H_5_)(C_5_(CF_3_)_5_)]**

In a dried 10 mL Schlenk flask [Fe(C_5_H_5_)(*o*DCB)][PF_6_] (0.17 g, 0.40 mmol, 1.0 equiv.) and [NEt_4_][C_5_(CF_3_)_5_] (0.21 g, 0.40 mmol, 1.0 equiv.) were dissolved in anhydrous *o*DFB (3 mL). The reaction mixture was stirred for 10 h at room temperature under visible light (470 nm) irradiation. Silica gel (200 mg) was added and the solvent was removed in high vacuum. The crude product loaded on silica was purified by column chromatography with *n*-pentane (first fraction). The organic fractions were combined and the solvent was removed under reduced pressure and the product was recrystallized from perfluorohexanes. The product [Fe(C_5_H_5_)(C_5_(CF_3_)_5_)] (0.12 g, 0.24 mmol) was obtained as a greenish crystalline solid with a yield of 60%.

**^1^H NMR** (400 MHz, CD_2_Cl_2_, rt) *δ* [ppm] = 4.94 (s, 5H). **^19^F NMR** (377 MHz, CD_2_Cl_2_, rt) *δ* [ppm] = −50.3 (s, 15F). **^13^C{^1^H} NMR** (151 MHz, CD_2_Cl_2_, rt) *δ* [ppm] = 78.1 (s, 5C). **^13^C{^19^F} NMR** (151 MHz, CD_2_Cl_2_, rt) *δ* [ppm] = 123.3 (m_c_, 5C), 112.5 (s, 5C). **FT-IR** (ATR) ṽ [cm^-1^] =3123 (w), 1485 (w), 1431 (s), 1140 (vs), 1009 (m), 953 (w), 875 (m), 854 (s), 811 (m), 750 (w), 710 (w), 656 (vs), 629 (s). **HRMS** (EI TOF, positive) m/z for [FeC_15_H_5_F_15_]^+^ calculated: 525.9501; measured: 525.9478. **EA** [FeC_15_H_5_F_15_] calculated: C: 34.25%, H: 0.96%; measured: C: 35.04%, H: 1.24. **UV/VIS** (rt): λ_max_= 407 nm. A molecular structure in solid state was measured for this compound (see Table S1 and Figure S20).

**Reaction S4: [Fe(C_5_H_5_)(C_5_(CF_3_)_5_)][AsF_6_]**

In a 8 mm PFA tube [Fe(C_5_H_5_)(C_5_(CF_3_)_5_)] (0.10 g, 0.19 mmol, 1.0 equiv.) was placed and cooled to −196 °C. SO_2_ (1 mL) and AsF_5_ (39 mg, 0.29 mmol, 1.5 equiv.) were condensed into the PFA tube. The reaction mixture was warmed to −20 °C and gently shaken at this temperature for 5 min. The volatiles were removed in high vacuum and the product [Fe(C_5_H_5_)(C_5_(CF_3_)_5_)][AsF_6_] (0.14 g, 0.19 mmol) was obtained as a deep green solid in quantitative yield.

**FT-IR** (ATR) ṽ [cm^-1^] = 3120 (w), 1595 (w), 1422 (w), 1207 (vs), 1153 (m), 910 (w), 880 (w), 703 (vs), 669 (s), 638 (s), 514 (m) . A molecular structure in solid state was measured for this compound (see Table S2 and Figure S21).

**Reaction S5: Oxidation with [Fe(C_5_H_5_)(C_5_(CF_3_)_5_)][AsF_6_]: [Fe(C_5_(CH_3_)_5_)_2_][AsF_6_]_2_**

In a 8 mm PFA tube [Fe(C_5_H_5_)(C_5_(CF_3_)_5_)][AsF_6_] (30 mg, 42 μmol, 2.0 equiv.) was placed and cooled to −196 °C. SO_2_ (0.5 mL) was condensed into the PFA tube and [Fe(C_5_(CH_3_)_5_)_2_] (6.9 mg, 21 μmol, 1.0 equiv.) was added at −196 °C. The reaction mixture was warmed to −20 °C and gently shaken at this temperature for 5 min. The volatiles were removed in high vacuum ([Fe(C_5_H_5_)(C_5_(CF_3_)_5_)] can be extracted with perfluorohexanes) and the residue was recrystallized from *a*HF. The product [Fe(C_5_(CH_3_)_5_)_2_][AsF_6_]_2_ was obtained as a brown crystalline solid.

The product was verified by single crystal X-ray diffraction and is consistent with literature.^[8]^

**Reaction S6: Oxidation with [Fe(C_5_H_5_)(C_5_(CF_3_)_5_)][AsF_6_]: 2,7-Dimethoxytriphenylene**

In a 10 mL Schlenk flask [Fe(C_5_H_5_)(C_5_(CF_3_)_5_)][AsF_6_] (50 mg, 70 μmol, 2.0 equiv.) was suspended in *o*DFB (2 mL) and 3,3′′-Dimethoxy-1,1′:2′,1′′-terphenyl (10 mg, 35 μmol, 1.0 equiv.) was added. The reaction mixture was stirred at room temperature for 1 h. Aqueous Na_2_SO_3_ (1.0 m, 10 mL) was added and the aqueous phase was extracted with CH_2_Cl_2_ (3 × 10 mL). The solvent was removed under reduced pressure ([Fe(C_5_H_5_)(C_5_(CF_3_)_5_)] can be extracted with perfluorohexanes) to give product 2,7-dimethoxytriphenylene (9.5 mg, 33 μmol) as a colourless amorphous solid with 95% yield.

**^1^H NMR** (400 MHz, CD_2_Cl_2_, rt) *δ* [ppm] = 8.61 (dd, ^3^*J*_H,H_ = 6.3, ^4^*J*_H,H_ = 3.4 Hz, 2H), 8.49 (d, ^3^*J*_H,H_ = 9.0 Hz, 2H), 8.04 (d, ^4^*J*_H,H_ = 2.6 Hz, 2H), 7.68 (dd, ^3^*J*_H,H_ = 6.3 Hz, ^4^*J*_H,H_ = 3.3 Hz, 2H), 7.27 (dd, ^3^*J*_H,H_ = 9.0 Hz, ^4^*J*_H,H_ = 2.6 Hz, 2H), 4.02 (s, 6H).The analytical data are consistent with those reported in literature.^[90]^

See Figure S13 and S14 for ^1^H and ^19^F NMR spectra before workup.

# NMR Spectra

**Figure S1.** ^1^H NMR (400 MHz, CD_2_Cl_2_, rt) spectrum of [NEt_4_][C_5_(CF_3_)_5_].


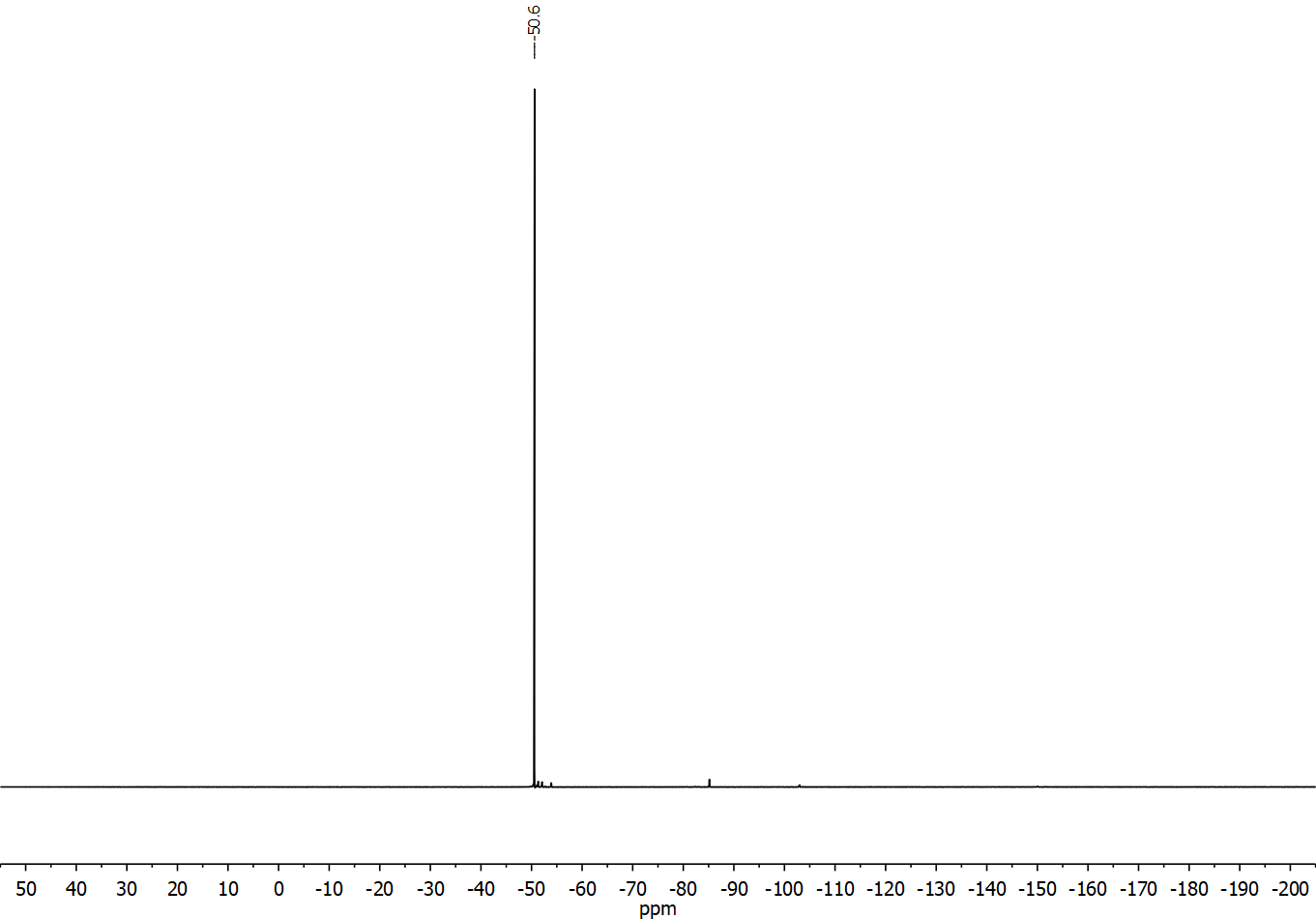


**Figure S2.** ^19^F NMR (377 MHz, CD_2_Cl_2_, rt) spectrum of [NEt_4_][C_5_(CF_3_)_5_].

**Figure S3.** ^13^C{^1^H} NMR (151 MHz, CD_2_Cl_2_, rt) spectrum of [NEt_4_][C_5_(CF_3_)_5_].

**Figure S4.** ^13^C{^19^F} NMR (151 MHz, CD_2_Cl_2_, rt) spectrum of [NEt_4_][C_5_(CF_3_)_5_].

**Figure S5.** ^1^H NMR (400 MHz, (CD_3_)_2_CO, rt) spectrum of [Fe(C_5_H_5_)(*o*DCB)][PF_6_].

**Figure S6.** ^19^F NMR (377 MHz, (CD_3_)_2_CO, rt) spectrum of [Fe(C_5_H_5_)(*o*DCB)][PF_6_].

**Figure S7.** ^31^P{^19^F} NMR (162 MHz, (CD_3_)_2_CO, rt) spectrum of [Fe(C_5_H_5_)(*o*DCB)][PF_6_].

**Figure S8.** ^13^C{^1^H} NMR (100 MHz, (CD_3_)_2_CO, rt) spectrum of [Fe(C_5_H_5_)(*o*DCB)][PF_6_].

**Figure S9.** ^1^H NMR (400 MHz, CD_2_Cl_2_, rt) spectrum of [Fe(C_5_H_5_)(C_5_(CF_3_)_5_)].

**Figure S10.** ^19^F NMR (377 MHz, CD_2_Cl_2_, rt) spectrum of [Fe(C_5_H_5_)(C_5_(CF_3_)_5_)].

**Figure S11.** ^13^C{^1^H} NMR (151 MHz, CD_2_Cl_2_, rt) spectrum of [Fe(C_5_H_5_)(C_5_(CF_3_)_5_)].

**Figure S12.** ^13^C{^19^F} NMR (151 MHz, CD_2_Cl_2_, rt) spectrum of [Fe(C_5_H_5_)(C_5_(CF_3_)_5_)].

**Figure S13.** ^1^H NMR (400 MHz, CD_2_Cl_2_, rt) spectrum before workup of reaction S6 with products 2,7-Dimethoxytriphenylene and [Fe(C_5_H_5_)(C_5_(CF_3_)_5_)].

**Figure S14.** ^19^F NMR (400 MHz, CD_2_Cl_2_, rt) spectrum before workup of reaction S6 with products 2,7-Dimethoxytriphenylene and [Fe(C_5_H_5_)(C_5_(CF_3_)_5_)].

# IR Spectra


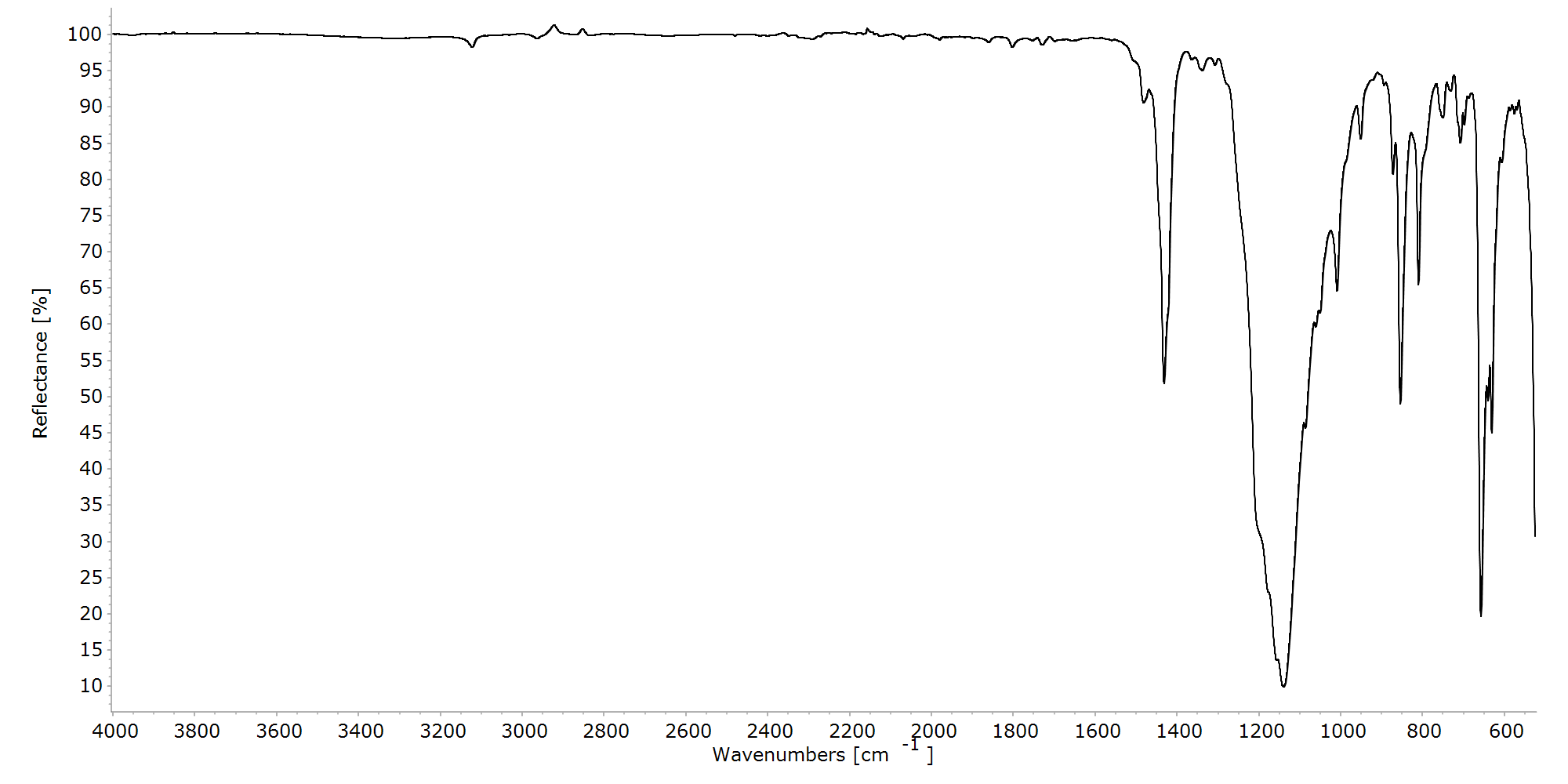


**Figure S15.** IR (ATR, rt) spectrum of [Fe(C_5_H_5_)(C_5_(CF_3_)_5_)].


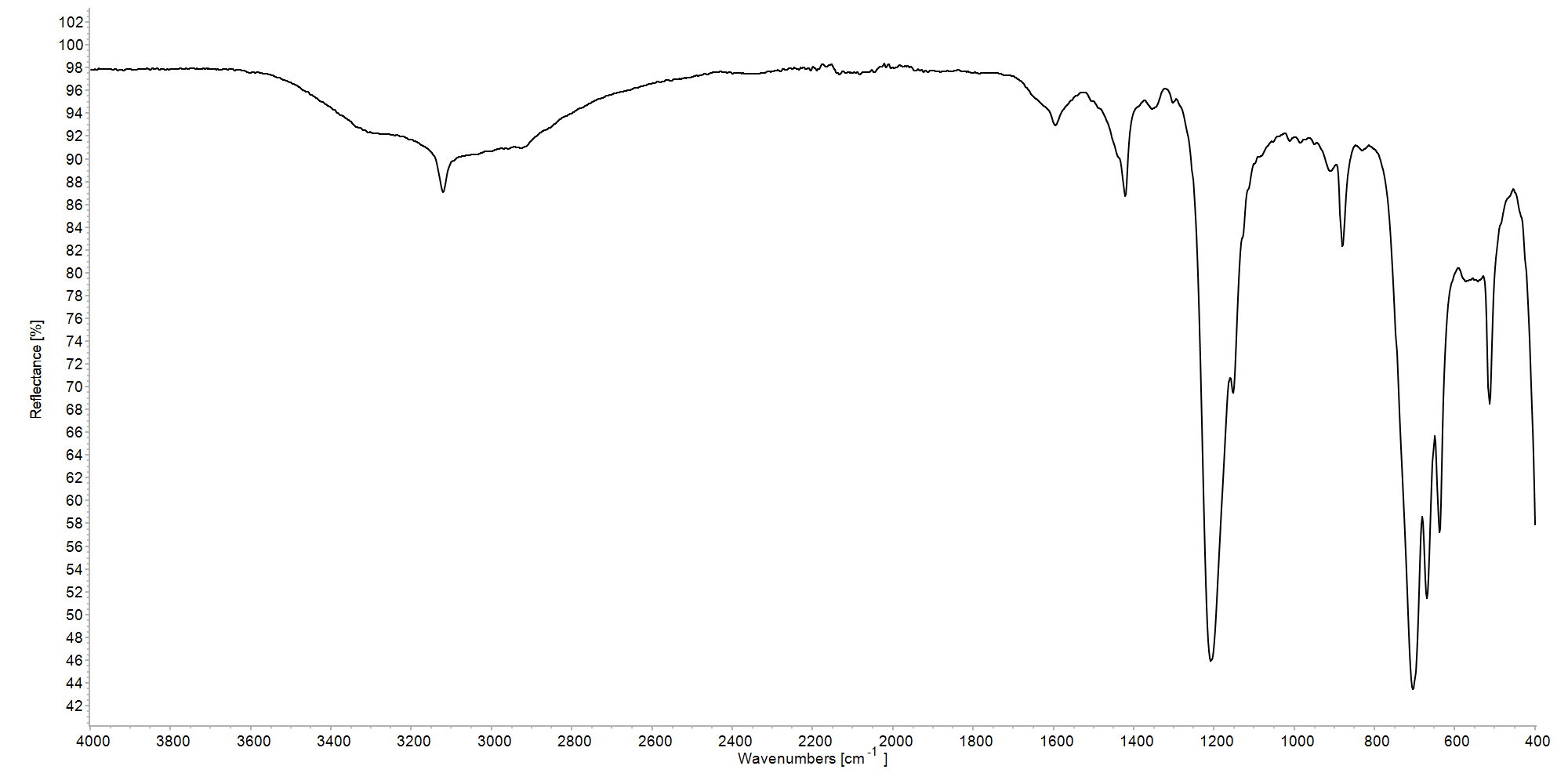


**Figure S16.** IR (ATR, rt) spectra of [Fe(C_5_H_5_)(C_5_(CF_3_)_5_)][AsF_6_].

# UV/VIS Spectra


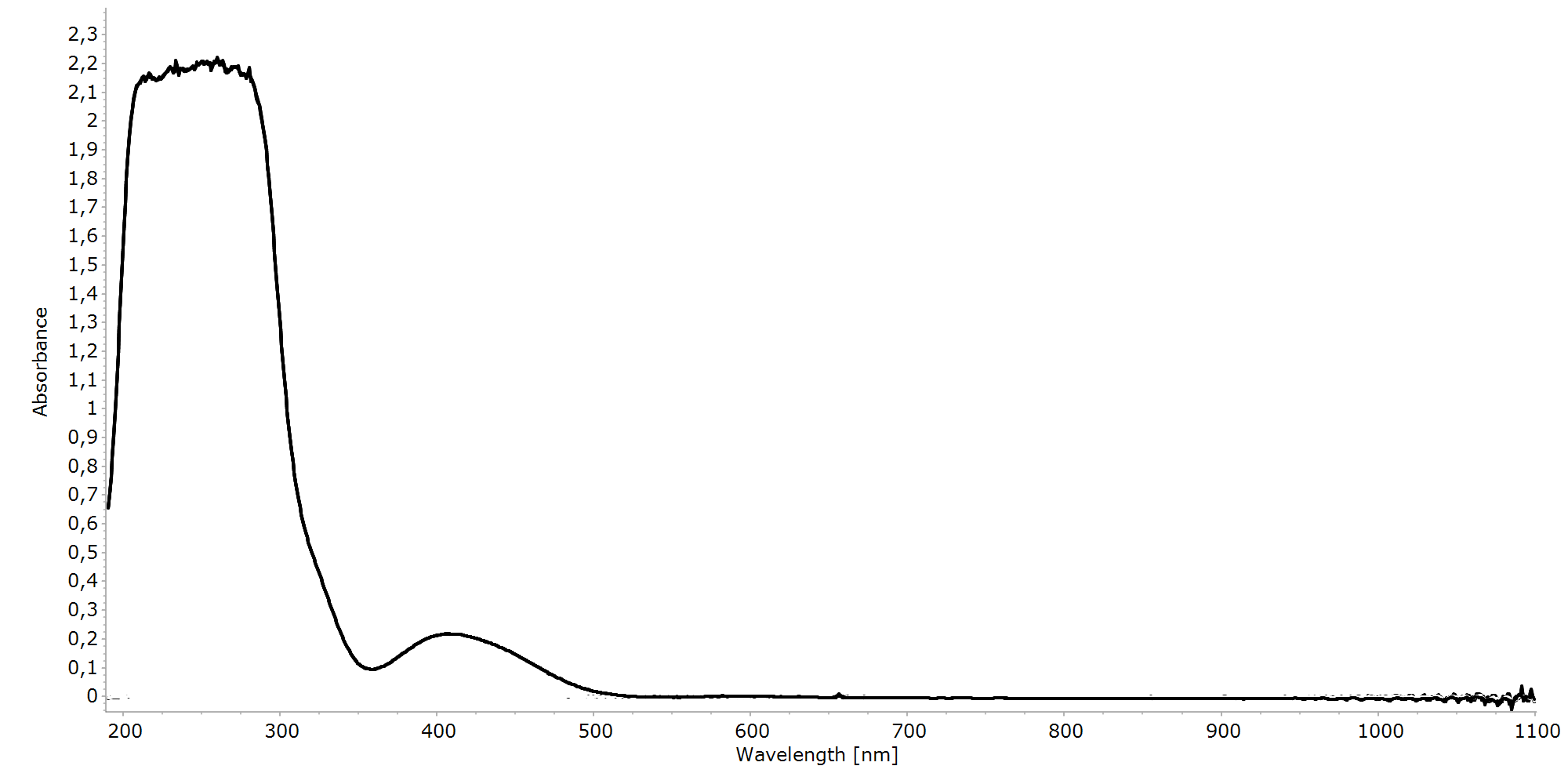


**Figure S17.** UV/VIS spectrum of [Fe(C_5_H_5_)(C_5_(CF_3_)_5_)].

# Cyclic Voltammograms


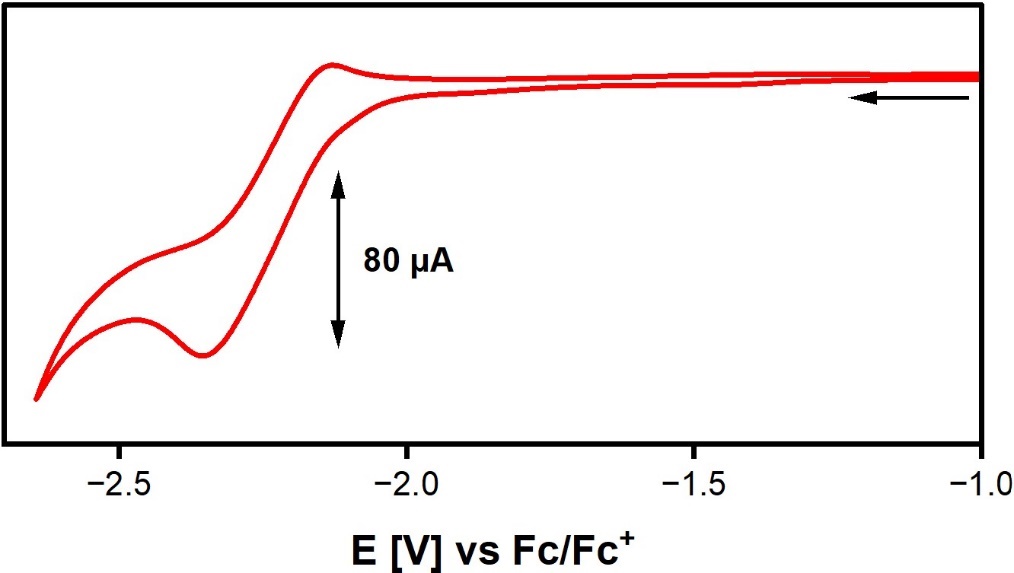


**Figure S18.** Cyclic voltammogram (rt, 100mV/s) of [Fe(C_5_H_5_)(C_5_(CF_3_)_5_)] showing a quasi-reversible reduction at *E*_1/2_ = -2.2 V in THF with [*n*Bu_4_N][PF_6_] as a supporting electrolyte.


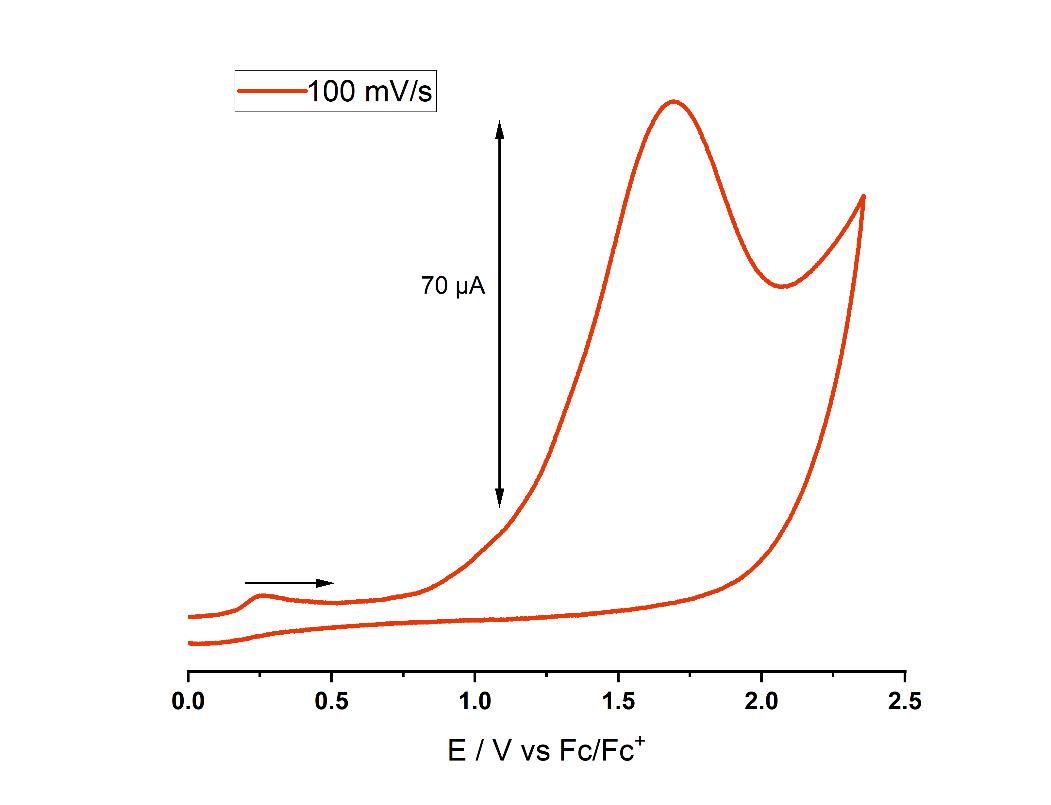


**Figure S19.** Cyclic voltammogram (rt, 100mV/s) of [Fe(C_5_H_5_)(C_5_(CF_3_)_5_)] showing irreversible oxidation in CH_2_Cl_2_ with [*n*Bu_4_N][PF_6_] as a supporting electrolyte.

# Crystallographic Data

| \| **Table S1.** Crystallographic data of [Fe(C_5_H_5_)(C_5_(CF_3_)_5_)]. \| \| \| --- \| --- \| \| Identification code \| 2429414 \| \| Empirical formula \| C_15_H_5_F_15_Fe \| \| Formula weight \| 526.04 \| \| Temperature/K \| 100 \| \| Crystal system \| monoclinic \| \| Space group \| *P*2_1_ \| \| a/Å \| 6.9340(3) \| \| b/Å \| 15.9012(8) \| \| c/Å \| 15.0081(8) \| \| α/° \| 90 \| \| β/° \| 90.024(2) \| \| γ/° \| 90 \| \| Volume/Å^3^ \| 1654.78(14) \| \| Z \| 4 \| \| ρ_calc_g/cm^3^ \| 2.111 \| \| μ/mm^‑1^ \| 1.073 \| \| F(000) \| 1024.0 \| \| Crystal size/mm^3^ \| 0.1 × 0.1 × 0.1 \| \| Radiation \| MoKα (λ = 0.71073) \| \| 2Θ range for data collection/° \| 3.732 to 55.8 \| \| Index ranges \| -9 ≤ h ≤ 9, -20 ≤ k ≤ 19, -19 ≤ l ≤ 19 \| \| Reflections collected \| 17774 \| \| Independent reflections \| 7300 [R_int_ = 0.0289, R_sigma_ = 0.0392] \| \| Data/restraints/parameters \| 7300/1/548 \| \| Goodness-of-fit on F^2^ \| 1.100 \| \| Final R indexes [I>=2σ (I)] \| R_1_ = 0.0446, wR_2_ = 0.1044 \| \| Final R indexes [all data] \| R_1_ = 0.0491, wR_2_ = 0.1098 \| \| Largest diff. peak/hole / e Å^-3^ \| 1.95/-0.78 \|   **Table S2.** Crystallographic data of [Fe(C_5_H_5_)(C_5_(CF_3_)_5_)][AsF_6_] ∙ 0.33 AsF_3_. | |
| --- | --- | --- | --- | --- | --- | --- | --- | --- | --- | --- | --- | --- | --- | --- | --- | --- | --- | --- | --- | --- | --- | --- | --- | --- | --- | --- | --- | --- | --- | --- | --- | --- | --- | --- | --- | --- | --- | --- | --- | --- | --- | --- | --- | --- | --- | --- | --- | --- | --- | --- | --- | --- | --- | --- | --- | --- | --- | --- | --- |
| Identification code | 2429415 |
| Empirical formula | C_30.25_As_2.67_F_45.54_Fe_2_ |
| Formula weight | 1540.11 |
| Temperature/K | 100.00 |
| Crystal system | hexagonal |
| Space group | *P*-6 |
| a/Å | 20.7577(9) |
| b/Å | 20.7577(9) |
| c/Å | 9.3497(7) |
| α/° | 90 |
| β/° | 90 |
| γ/° | 120 |
| Volume/Å^3^ | 3488.9(4) |
| Z | 3 |
| ρ_calc_g/cm^3^ | 2.199 |
| μ/mm^‑1^ | 2.717 |
| F(000) | 2194.0 |
| Crystal size/mm^3^ | 0.1 × 0.1 × 0.1 |
| Radiation | MoKα (λ = 0.71073) |
| 2Θ range for data collection/° | 3.924 to 58.31 |
| Index ranges | -28 ≤ h ≤ 28, -28 ≤ k ≤ 28, -12 ≤ l ≤ 12 |
| Reflections collected | 155211 |
| Independent reflections | 6596 [R_int_ = 0.0406, R_sigma_ = 0.0136] |
| Data/restraints/parameters | 6596/2/416 |
| Goodness-of-fit on F^2^ | 1.042 |
| Final R indexes [I>=2σ (I)] | R_1_ = 0.0866, wR_2_ = 0.2306 |
| Final R indexes [all data] | R_1_ = 0.0903, wR_2_ = 0.2343 |
| Largest diff. peak/hole / e Å^-3^ | 1.17/-1.21 |
| Flack parameter | -0.0084(18) |


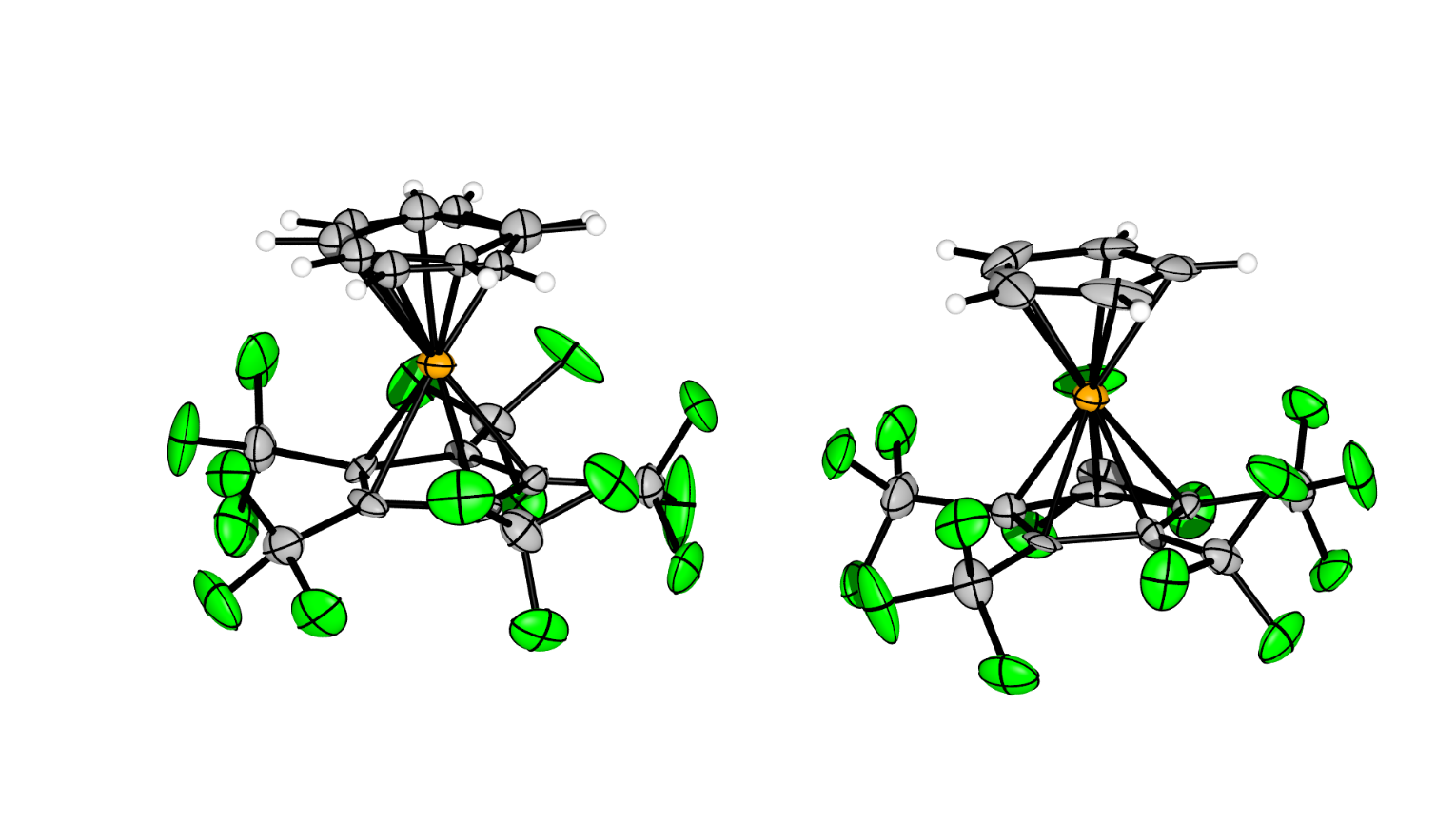


**Figure S20.** Molecular structure in solid state of [Fe(C_5_H_5_)(C_5_(CF_3_)_5_)]. Ellipsoids are depicted with 50% probability level. Colour code: white-hydrogen, grey-carbon, green-fluorine, orange-iron.


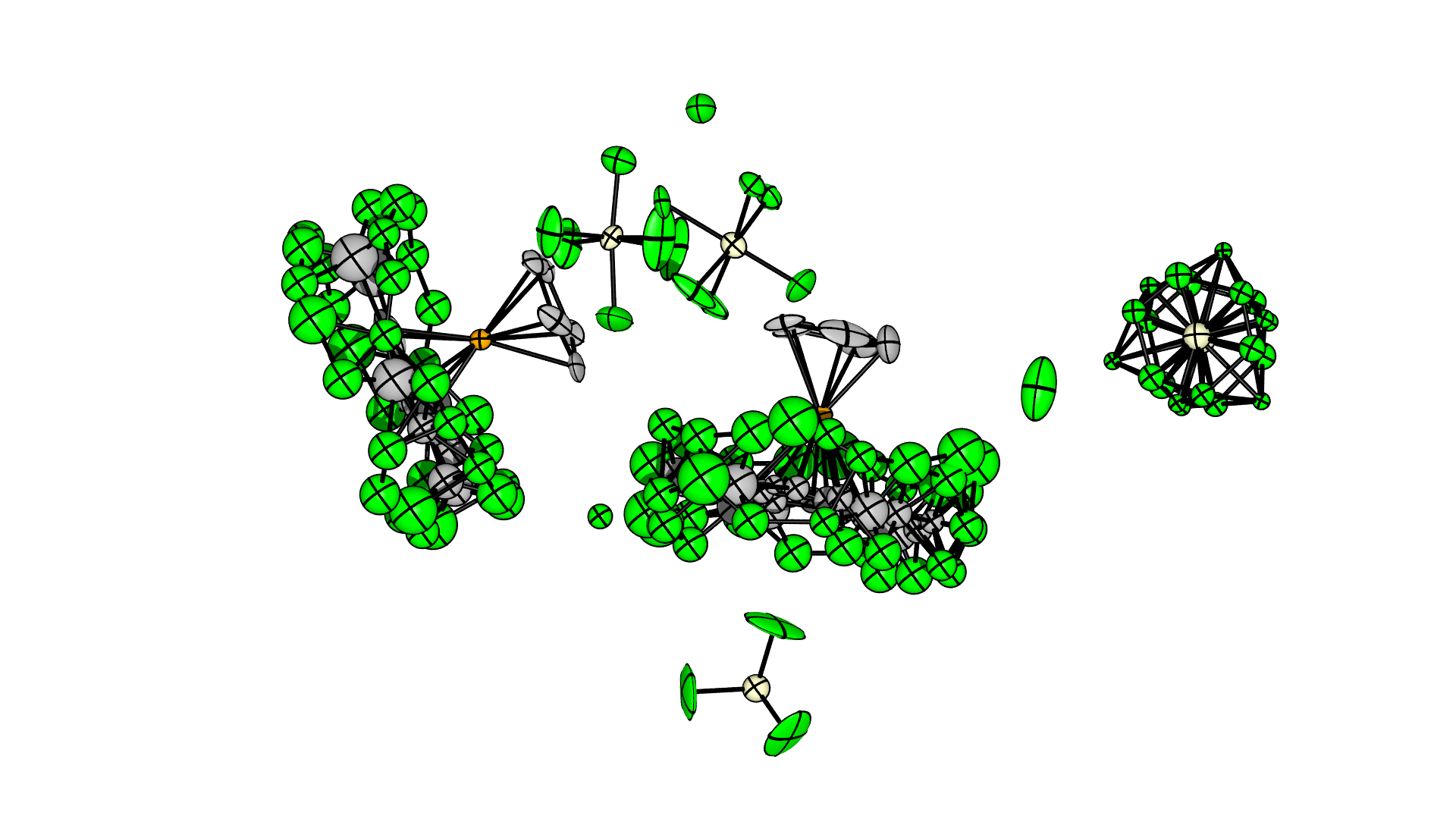


**Figure S21.** Molecular structure in solid state of [Fe(C_5_H_5_)(C_5_(CF_3_)_5_)][AsF_6_] ∙ 0.33 AsF_3_. Ellipsoids are depicted with 50% probability level. Colour code: white-hydrogen, grey-carbon, green-fluorine, orange-iron, beige-arsenic.^[a]^

[a] Indexing yields hexagonal unit cell with 3916 out of 5543 reflections assigned. No second domain could be identified. E statistics indicates centro-symmetric space group type, however, racemic mixture is observed according to significantly increased refinement values in non-centrosymmetric space group in combination with inversion twin refinement (-1 0 0 0 -1 0 0 0 1 with BASF 0.51(4)).

Heavy disorder of the [C_5_(CF_3_)_5_]^−^ moieties and AsF_3_ units is observed, both in hexagonal and triclinic space group types. Assignment of proton positions unreliable due to disorder. Non-connected F-atoms (F23, occ. 0.25; F45, occ. 0.5; F6, occ. 1) assumed to be solvate HF.

Rotational disorder of central C_5_-rings at Fe1 yields 10 positions with occupation 0.5, connected to 5 carbon atom from CF_3_ groups on 14 positions with occupations 0.5 or 0.25 (yielding five carbon atoms total for CF_3_ groups). Due to additional rotational freedom of fluoride atoms in CF_3_ groups, assuming minimum disorder of fluoride on two positions each, at least 84 fluoride positions would be expected, out of which only 39 could be refined based on the experimental Fourier synthesis.

A similar disorder is assumed for ligands at Fe2, however, no stable refinement could be observed for split positions at C_5_ ring comprising CF_3_ substitution with parallel refinement of fluoride atoms. Best refinement results were obtained for five distinct positions of central C_5_ ring and eight positions for carbon atoms of CF_3_ substituent, totaling five carbon atoms from the sum of occupation factors each. Residual density clearly indicates more split positions, which do not yield stable refinements. Out of 84 fluoride atom positions (vide supra), only 34 could be refined reliably.

[AsF_6_]^−^ moiety comprises small disorder of fluoride ions on two positions each, which results in inferior refinement values. Single positions were chosen instead, yielding comparably large anisotropic displacement parameters. AsF_3_ moiety of As3 assumes two split positions with an occupation of 0.5 each. Each connected fluoride substituent is disordered on three positions each, yielding a total of 18 fluoride positions with a total occupation of 24/8.

AsF_3_ moiety of As2 assumes two split positions with an occupation of 0.5 each. Corresponding six fluoride positions are fixed at 0.5 occupation, however, additional six disorder positions are indicated by max. residual density (1.0 e/A³). No stable model could be refined comprising the total 12 positions with a physically meaningful occupation number combination.

Solvent mask was employed for residual void comprising unassignable electron density (approx. 20 A³ with a total of 6 electrons. R1/wR2/GooF with solvent mask and without, respectively: 8.70/22.68/1.071, 9.41/25.29/1.055.

# Quantum Chemical Calculations

**Table S3.** Results of energy decomposition analysis of the Fe−Cp and Fe−X interaction in [Fe(C_5_H_5_)X] (X = [C_5_H_5_]^−^, [C_5_F_5_]^−^, [C_5_(CF_3_)_5_]^−^) at the BP86+D4/TZ2P//r^2^SCAN-3c level of theory in kJ/mol.

| Fragment 1 | Fragment 2 | ΔE_Pauli_ | ΔE_Elstat._ | ΔE_Orb.Int_ | ΔE_COSMO_^[a]^ | ΔE_Int._ |
| --- | --- | --- | --- | --- | --- | --- |
| [Fe(C_5_H_5_)]^+^ | [C_5_H_5_]^−^ | 748.6 | −1072.9 | −694.8 | 492.8 | −554.6 |
| [Fe(C_5_F_5_)]^+^ | [C_5_H_5_]^−^ | 737.6 | −1111.5 | −715.3 | 544.2 | −572.3 |
| [Fe(C_5_(CF_3_)_5_)]^+^ | [C_5_H_5_]^−^ | 752.8 | −1148.0 | −808.6 | 549.1 | −697.7 |
| [Fe(C_5_(CF_3_)_5_)]^+^ | [C_5_H_5_]^− [b]^ | 796.3 | −1170.9 | −828.7 | 549.3 | −697.6 |
| [Fe(C_5_H_5_)]^+^ | [C_5_F_5_]^−^ | 850.6 | −985.8 | −823.4 | 434.1 | −558.5 |
| [Fe(C_5_H_5_)]^+^ | [C_5_(CF_3_)_5_]^−^ | 782.0 | −801.3 | −733.5 | 379.9 | −433.0 |

^[a]^ Contribution only includes the electrostatic interaction energy with the cavity charges. ^[b]^Calculations were performed at a Fe-(C_5_H_5_) distance corresponding to [Fe(C_5_H_5_)_2_].

**Table S4.** (Summed) eigenvalues of the most important contributions in an ETS-NOCV analysis of the Fe-Cp and Fe-X interaction in [Fe(C_5_H_5_)X] (X=[C_5_H_5_]^−^, [C_5_F_5_]^−^, [C_5_(CF_3_)_5_]^−^) group according to their symmetry at the BP86+D4/TZ2P//r^2^SCAN-3c level of theory in kJ/mol.

| Fragment 1 | Fragment 2 | ΔE_NOCV,σ_ | ΔE_NOCV,π_ | ΔE_NOCV,δ_ |
| --- | --- | --- | --- | --- |
| [Fe(C_5_H_5_)]^+^ | [C_5_H_5_]^−^ | 82.7 | 425.4 | 125.8 |
| [Fe(C_5_F_5_)]^+^ | [C_5_H_5_]^−^ | 84.8 | 458.9 | 102.4 |
| [Fe(C_5_(CF_3_)_5_)]^+^ | [C_5_H_5_]^−^ | 95.7 | 542.5 | 91.0 |
| [Fe(C_5_(CF_3_)_5_)]^+^ | [C_5_H_5_]^− [a]^ | 96.9 | 552.1 | 97.4 |
| [Fe(C_5_H_5_)]^+^ | [C_5_F_5_]^−^ | 128.5 | 461.5 | 156.1 |
| [Fe(C_5_H_5_)]^+^ | [C_5_(CF_3_)_5_]^−^ | 92.8 | 369.8 | 189.6 |

^[a]^ Calculations were performed at a Fe-(C_5_H_5_) distance corresponding to [Fe(C_5_H_5_)_2_].

**Table S5.** Calculated electrochemical potentials vs. ferrocene/ferrocenium electrode at the ωB97X-D4/def2-QZVPPD//r^2^SCAN-3c (COSMO-RS) level^[a]^ in V vs. Fc/Fc^+^.^[29]^

|  | ΔE_calc._ | ΔE_exp._ |
| --- | --- | --- |
| [Fe(C_5_H_5_)(C_5_H_4_F)] | 0.19 | 0.14 |
| [Fe(C_5_H_5_)(C_5_H_3_F_2_)] | 0.31 | 0.28 |
| [Fe(C_5_H_5_)(C_5_H_2_F_3_)] | 0.47 | 0.42 |
| [Fe(C_5_H_5_)(C_5_HF_4_)] | 0.64 | 0.62 |
| [Fe(C_5_H_5_)(C_5_F_5_)] | 0.77 | 0.82 |
| [Fe(C_5_H_5_)(C_5_(CF_3_)_5_)] | 1.45 | 1.35^[b]^ |

^[a]^ From computed free energies in solution. ^[b]^ This work.


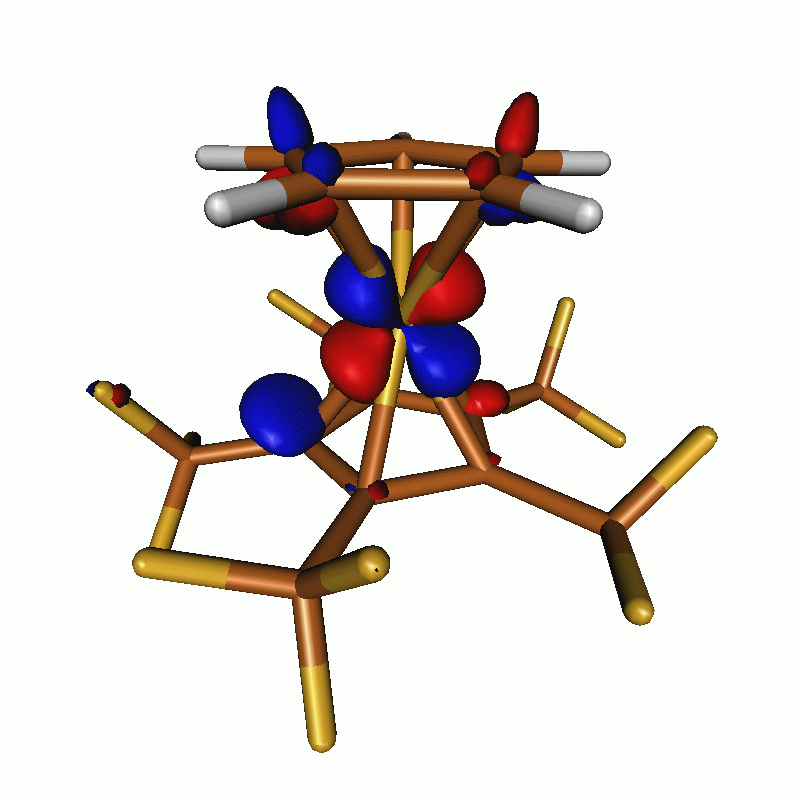

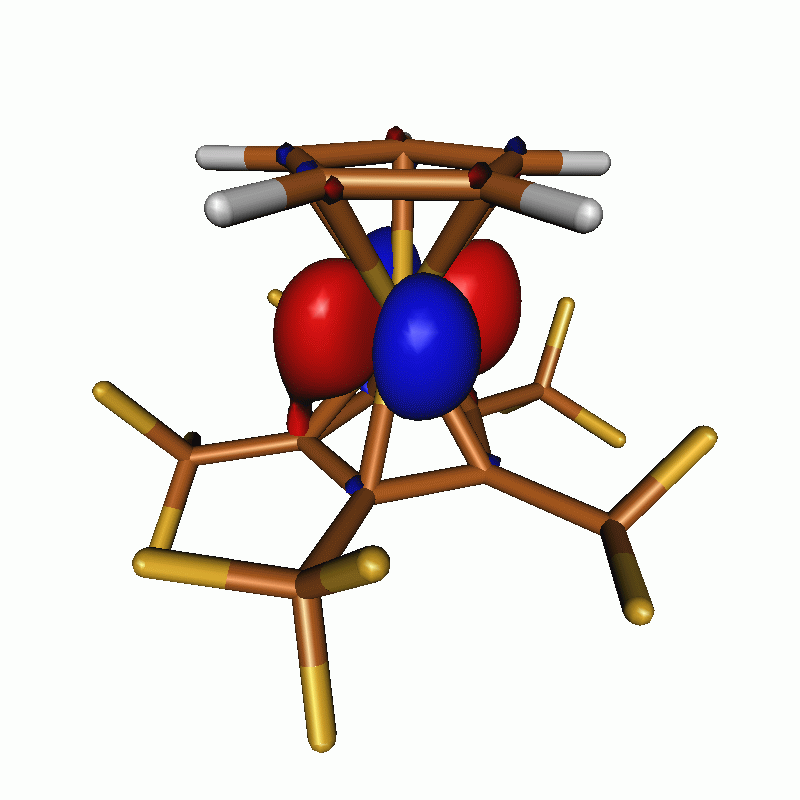


a)

b)

**Figure S22.** Frontier orbitals of [Fe(C_5_H_5_)(C_5_(CF_3_)_5_)]: a) HOMO and b) LUMO at the ωB97X-D4/def2-QZVPPD//r^2^SCAN-3c/COSMO level. Orbitals are shown as isovalue surfaces at a value of 0.05.

# References

[65] H. E. Gottlieb, V. Kotlyar, A. Nudelman, *J. Org. Chem.* **1997**, *62*, 7512-7515.

[66] G. R. Fulmer, A. J. M. Miller, N. H. Sherden, H. E. Gottlieb, B. M. Stoltz, J. E. Bercaw, K. I. Goldberg, *Organometallics* **2010**, *29*, 2176-2179.

[67] R. K. Harris, E. D. Becker, S. M. Cabral de Menezes, R. Goodfellow, P. Granger, *Pure Appl. Chem.* 2001, *73*, 1795-1818.

[68] M. R. Willcott, *J. Am. Chem. Soc.*, 2009, **131**, 13180.

[69] O. V. Dolomanov, L. J. Bourhis, R. J. Gildea, J. A. K. Howard, H. Puschmann, *J. Appl. Cryst.*, 2009, **42**, 339-341.

[70] G. M. Sheldrick, *Acta Cryst*., 2015, **A71**, 3-8.

[71] G. M. Sheldrick, *SHELXL Version 2014/7, Program for Crystal Structure Solution and Refinement*; Göttingen, Germany, 2014.

[72] G. M. Sheldrick, *Acta Cryst.*, 2008, **A64**, 112-122.

[73] K. Brandenburg, Diamond: Crystal and Molecular Structure Visualization http://www.crystalimpact.com/diamond.

[74] Persistence of Vision Pty. Ltd. Persistence of Vision Raytracer. Ltd., Persistence of Vision Pty. 2004.

[75] S. Grimme, A. Hansen, S. Ehlert, J.-M. Mewes, *J. Chem. Phys.* **2021**, *154*, 064103.

[76] F. Neese, Wiley Interdiscip. Rev. *Comput. Mol. Sci.* **2018**, *8*, e1327.

[77] A. Najibi, L. Goerigk, *J. Comput. Chem.* **2020**, *41*, 2562-2572.

[78] D. Rappoport, F. Furche, *J. Chem. Phys.* **2010**, *133*, 134105.

[79] F. Neese, F. Wennmohs, A. Hansen, U. Becker, *Chem. Phys.* **2009**, *356*, 98-109.

[80] T. Gerlach, S. Müller, A. G. Castilla, I. Smirnova, *Fluid Ph. Equilib.* **2022**, *560*, 113472.

[81] S. Grimme, *Chem. Eur. J.* **2012**, *18*, 9955-9964.

[82] A. D. Becke, *Phys. Rev. A* **1988**, *38*, 3098-3100.

[83] J. P. Perdew, *Phys. Rev. B*, **1986**, *33*, 8822-8824.

[84] J. P. Perdew, *Phys. Rev. B*, **1986**, *34*, 7406.

[85] E. Caldeweyher, S. Ehlert, A. Hansen, H. Neugebauer, S. Spicher, C. Bannwarth, S. Grimme, *J. Chem. Phys.* **2019**, *150*, 154122.

[86] G. te Velde, F. M. Bickelhaupt, E. J. Baerends, C. Fonseca Guerra, S. J. A. van Gisbergen, J. G. Snijders, T. Ziegler, *J. Comput. Chem.* **2001**, *22*, 931-967.

[87] AMS 2023.1, SCM, Theoretical Chemistry, Vrije Universiteit, Amsterdam, The Netherlands, http://www.scm.com.

[88] R. D. Chambers, W. K. Gray, J. F. S. Vaughan, S. R. Korn, M. Médebielle, A. S. Batsanov, C. W. Lehmann, J. A. K. Howard, *J. Chem. Soc.* *Perkin Trans. 1*, **1997**, 135-146.

[89] C. Malchau, N. Ultes, J. Ehrhard, T. R. Eger, D. V. Fries, B. Oelkers, S. Becker, G. Niedner Schatteburg, W. R. Thiel, *J. Organomet. Chem.* **2023**, *987*, 122618.

[90] B. J. King, J. Kroulík, C. R. Robertson, P. Rempala, C. L. Hilton, J. D. Korinek, L. M. Gortari, *J. Org. Chem.* **2007**, *72*, 2279-2288.
